# Oxidative Stress Induced Mitochondrial Protein Kinase A Mediates Cytochrome C Oxidase Dysfunction

**DOI:** 10.1371/journal.pone.0077129

**Published:** 2013-10-10

**Authors:** Satish Srinivasan, Joseph Spear, Karunakaran Chandran, Joy Joseph, Balaraman Kalyanaraman, Narayan G. Avadhani

**Affiliations:** 1 Department of Animal Biology and the Mari Lowe Center for Comparative Oncology, School of Veterinary Medicine, University of Pennsylvania, Philadelphia, Pennsylvania, United States of America; 2 Department of Biophysics, and Center for Free Radical Biology, Medical College of Wisconsin, Milwaukee, Wisconsin, United States of America; University of Texas Health Science Center at San Antonio, United States of America

## Abstract

Previously we showed that Protein kinase A (PKA) activated in hypoxia and myocardial ischemia/reperfusion mediates phosphorylation of subunits I, IVi1 and Vb of cytochrome c oxidase. However, the mechanism of activation of the kinase under hypoxia remains unclear. It is also unclear if hypoxic stress activated PKA is different from the cAMP dependent mitochondrial PKA activity reported under normal physiological conditions. In this study using RAW 264.7 macrophages and *in vitro* perfused mouse heart system we investigated the nature of PKA activated under hypoxia. Limited protease treatment and digitonin fractionation of intact mitochondria suggests that higher mitochondrial PKA activity under hypoxia is mainly due to increased sequestration of PKA Catalytic α (PKAα) subunit in the mitochondrial matrix compartment. The increase in PKA activity is independent of mitochondrial cAMP and is not inhibited by adenylate cyclase inhibitor, KH7. Instead, activation of hypoxia-induced PKA is dependent on reactive oxygen species (ROS). H89, an inhibitor of PKA activity and the antioxidant Mito-CP prevented loss of CcO activity in macrophages under hypoxia and in mouse heart under ischemia/reperfusion injury. Substitution of wild type subunit Vb of CcO with phosphorylation resistant S40A mutant subunit attenuated the loss of CcO activity and reduced ROS production. These results provide a compelling evidence for hypoxia induced phosphorylation as a signal for CcO dysfunction. The results also describe a novel mechanism of mitochondrial PKA activation which is independent of mitochondrial cAMP, but responsive to ROS.

## Introduction

Hypoxic environment characterized by low O_2_ tension is encountered by cells and tissues under many pathological conditions including ischemia, inflammation, skeletal-muscular disorders, and solid tumors. A large part of respired O_2_ in metazoan organisms is utilized by mitochondria in processes directly linked to oxidative phosphorylation. Not surprisingly, mitochondria undergo a time dependent progressive damage during cellular hypoxia and myocardial ischemia/reperfusion. The mitochondrial electron transport chain is one of the principal targets of hypoxia induced oxidative damage [1]. While complexes I and V are affected relatively early, prolonged ischemia damages complexes III and IV [2–7]. The mechanisms by which hypoxia affects different electron transport chain complexes appears to differ with respect to each complex. Activity of Complex I decreases likely due to oxidative loss of flavin mononucleotide coenzyme [2,8]. Complex III activity is affected due to inactivation of Fe-S protein [6]. Complex V activity has been shown to be reversibly inhibited by changes in pH during hypoxia [8]. Our previous study showed that Complex IV activity is decreased due to phosphorylation related degradation of specific subunits of the complex [9,10].

Protein kinases play an important role in the modulation of oxidative phosphorylation under different physiological and environmental conditions [11]. Phosphorylation of mitochondrial proteins has been reported both as part of regulatory mechanism as well as stress response [12–16]. Various cytosolic kinases such as cAMP dependent protein kinase (PKA), Protein kinase C isoforms PKCδ and PKCε, Akt and Glycogen synthase kinase 3β (GSK 3β) have been shown to be present in the mitochondrial inner membrane-matrix compartment and their role in phosphorylation of mitochondrial proteins including the electron transport chain complexes have been reported over the years [17–20]. Of the five electron transport chain complexes, maximum number of phosphorylation sites has been predicted for cytochrome c oxidase, complex IV (CcO) [21]. So far, eighteen different sites of CcO have been experimentally shown to be phosphorylated under physiological and pathological conditions [21]. Depending on the residues and subunits involved, some of these phosphorylations have been shown to either activate or inhibit CcO enzyme activity [22–24]. PKA has been shown to be the major kinase acting on CcO with seven experimentally tested phosphorylation sites [9,10,22–25]. Subunits I, II, and Vb of CcO are phosphorylated *in vitro* by PKA [23]. Notably, these phosphorylation resulted in inhibition of CcO activity by 40–70%, possibly by increasing ATP dependent allosteric inhibition [23]. Similarly, Theophylline treatment resulted in phosphorylation of Tyr304 of subunit I in a cAMP dependent manner [24]. This phosphorylation also inhibited enzyme activity [24]. On the other hand, phosphorylation of S58 of subunit IVi1 by PKA increased CcO activity under normoxic conditions, possibly by abolishing allosteric inhibition [22]. 

In our previous studies we showed that PKA plays a major role in both hypoxia and ischemia-reperfusion mediated injury to the electron transport chain [10]. Our results showed that, three of the subunits of CcO, namely, subunits I, IVi1 and Vb are phosphorylated under hypoxia and that the phosphorylation could be abolished by H89, an inhibitor of PKA activity [10]. Furthermore, phosphorylation resulted in preferential degradation of these subunits leading to loss of CcO activity [10]. Moreover, inhibition of PKA activity using H89 or PKI, a specific peptide inhibitor of PKA, prevented the hypoxia-mediated loss of CcO subunits and activity. Phosphorylation of CcO, both in cells grown under hypoxic conditions, as well as, in rabbit hearts subjected to global or focal ischemia resulted in significantly reduced CcO activity and increased production of reactive O_2_ species (ROS) in an in vitro reconstituted system [10]. Further, we mapped the phosphorylation sites of these three subunits using a nano-LC–MS/MS system and showed that two tandem Ser residues (Ser115/Ser116) of subunit I, Thr/Ser52 of subunit IVi1 and Ser40 of subunit Vb are phosphorylated [9]. The phosphorylated residues were undetectable in CcO subunits from control hearts as well as those pretreated with H89, suggesting that the phosphorylation was associated with ischemia/reperfusion injury and is possibly dependent on PKA activity [9].

While phosphorylation of CcO subunits during hypoxia or myocardial ischemia-reperfusion has been shown to be important in causing mitochondrial and cellular injury, so far there has been no direct evidence to show the role of PKA and its mitochondrial activation under hypoxia. Additionally, since reports from various groups show that subunit phosphorylation may result in both inhibition and activation of CcO under different pathophysiological conditions, it was imperative to investigate the distinctive nature of the kinase activated under hypoxia and myocardial ischemia. In this paper we show an increased sequestration of PKAα subunit in mitochondrial matrix compartment under hypoxia with an accompanying increase in mitochondrial PKA activity by a process that is linked to ROS formation. We propose that the increased mitochondrial matrix PKA under hypoxia and myocardial ischemia mediates the loss of CcO subunits and activity. Finally, we show that replacement of wild type Vb subunit of CcO with phosphorylation resistant mutant reduces hypoxia-induced degradation and loss of activity. 

## Materials and Methods

### Cell culture and treatments

RAW 264.7 macrophages (ATCC, Manassas, VA) and H9C2 cardiomyocytes were maintained in DMEM (Dulbecco's modified Eagle's medium; Invitrogen) supplemented with 10% FBS. Cells at 70–80% confluence were grown under hypoxic (1% O_2_) or normoxic (21% O_2_) conditions for indicated durations. For hypoxia, growth medium was pre-equilibrated at 1% O2 in hypoxia glove box (Coy Laboratories, MI) overnight before adding to the cells. Cells were maintained under hypoxia for specified lengths of time. Harvesting and fractionation of cells at the end of hypoxia were performed at normoxic conditions. H89, MPI, KH7, N-Acetyl Cysteine (NAC) and Mito-CP were added at indicated concentrations to cells at the start of hypoxia inside the hypoxia chamber. Mito-CP is Mito-carboxy proxyl and is a mitochondria targeted nitroxide that mimics superoxide dismutase activity [26]. 

### Isolation of mitochondria

Mitochondria were prepared by differential centrifugation as described previously [27]. Briefly, cells were washed with cold Phosphate buffered saline and homogenized with a Dounce glass homogenizer in H-medium (70 mM sucrose, 220 mM mannitol, 2.5 mM HEPES, pH 7.4, 2 mM EDTA containing 1mM PMSF, 1 µg/ml each of Pepstatin, Leupeptin, Aprotinin and Antipain). Mouse heart tissues were finely minced and disrupted with two pulses of 5 seconds each in a polytron tissue homogenizer. The crude extract was then homogenized in a Dounce glass homogenizer as described before. Subcellular fractions were prepared by differential centrifugation. The mitochondrial pellet obtained was resuspended in H-medium and layered on 0.8M Sucrose and centrifuged at 10,000Xg for 15min at 4°C to remove any cytosolic contamination. The resulting pellet was washed once with H-medium and the protein concentration of the final mitochondrial suspension in H-medium was determined according to the method of Lowry et al [28].

### Mitochondrial localization of PKA

#### Digitonin fractionation

Isolated mitochondria (100 µg) were resuspended in H-medium (210mM Mannitol, 70mM Sucrose, 10mM HEPES, 1mM EDTA, pH 7.3), and incubated with increasing concentrations of digitonin (0% to 1% of mitochondrial protein) in the presence of trypsin 15 µg. Reactions were carried out for 20 min on ice, after which, 150 µg soybean trypsin inhibitor was added to stop trypsin action. Samples were then centrifuged at 20,000 *g* for 10 min at 4°C. The pellets were washed once with H-medium and resuspended in Laemmli’s buffer. Further processing of the normoxic and hypoxic samples were done simultaneously and under identical conditions as explained below. Both normoxic and hypoxic samples were separated on 12% SDS-polacrylamide gels, run simultaneously in a Biorad Mini Protean Tetracell system (Biorad, CA). The separated proteins were transferred onto nitrocellulose membranes. Incubation with antibodies against CcO IVi1 (Abcam, Cambridge, MA), PKAα (SantaCruz, CA) and TOM20 (SantaCruz) and subsequently with secondary antibodies conjugated to either IR680 or IR800 dyes (Licor) were done simultaneously for both normoxic and hypoxic protein blots under identical conditions. To maintain uniform exposure, the blots were scanned in the Odyssey Licor system (Licor biotechnology, Lincoln, NE) at an intensity setting of 3.5 for 800nm and 2.5 for 680nm. The figure is a representative blot from 3 separate experiments.

#### Ectopic expression of PKAα

COS-7 cells grown on coverslips were transfected with cDNA coding for PKAα fused to a Myc-DDK tag at its C-terminus. Thirty six hours after transfection, the cells were maintained under either normoxia or hypoxia (1% O_2_) for 12h. At the end of hypoxia, the cells were fixed with cold methanol for 10min and rapidly washed with ice cold PBS. Antibodies against FLAG and CcO subunit I (Abcam, Cambridge, MA), were used to stain the cells. Alexa 488 and 594 conjugated antibodies (Life technologies, Grand Island, NY) were used as secondary antibodies.

### Mouse heart ischemia-reperfusion protocol

All the mouse work was carried out in compliance with the institutional guidelines and regulatory standards of University of Pennsylvania. This study was approved by the Institutional Animal Care and Use Committee (IACUC, Protocol No: 802781) of University of Pennsylvania. Swiss albino mice (25-30 g) were heparinized (2000 U/kg IP) and then anesthetized with avertin (100 mg/kg IP). The hearts were rapidly removed and perfused with an oxygenated Krebs-Henseleit solution containing 120mM NaCl, 5.8mM KCl, 25mM NaHCO_3_, 1.2mM NaH_2_PO_4_, 1.2mM MgSO_4_, 1mM CaCl_2_, and 10mM dextrose, pH 7.4, at 37°C in a Langendorff coronary perfusion system. Hearts were submerged into a heat-jacketed organ bath set at 37°C. After 20 minutes of equilibration, global ischemia was initiated by stopping coronary perfusion. Hearts were subjected to a 30-minute global ischemia and a 120-minute full reperfusion. For different treatments, the modulating chemical was included in the perfusion medium at the following concentrations: H89 (1µM) (n=6), MitoQ (0.1 µM) (n=3) and Mito CP (1µM) (n=3). At the end of the reperfusion period, hearts were sliced into 1-mm-thick transverse sections and incubated in triphenyltetrazolium chloride solution (1% in phosphate buffer, pH 7.4) at 37°C for 15 minutes as reported previously [29]. Infarct size was expressed as a ratio of necrotic area over total area at risk. 

### Protein kinase A assay

PKA assay was performed using a kit as described by the manufacturer (PKA assay kit, 17-134, Millipore). The assay uses Kemptide, a highly specific PKA substrate. To account for PKA independent phosphorylation, for each assay a parallel reaction was carried out in presence of PKI, a specific PKA inhibitor. The final PKA activity was obtained by subtracting residual activity in presence of PKI from total activity. Sample extracts for the assay were prepared by lysing either whole cells or isolated mitochondria in lysis buffer containing 50mM Tris-HCl, 1% NP-40, 150mM NaCl and 1mM EDTA. The kinase reaction was carried out for 30 min at 30°C in a total volume of 60 µl and 10 µg of protein as enzyme source. After the reaction, the assay mix was spotted on P80 phospho cellulose paper, washed and counted in a Beckman model LS5000TA liquid scintillation counter.

### SDS and blue native PAGE

For SDS/PAGE analysis, either 40 μg of mitochondrial extract or 80 μg of whole cell extract was separated on a 12% denaturing polyacrylamide gel and transferred to nitrocellulose membranes (Bio-Rad laboratories, CA).

Blue native PAGE was carried out essentially as described previously [30]. Briefly, 150 μg of mitochondrial protein was solubilized in 30 μl of solubilization buffer containing 1.5 M aminocaproic acid, 50 mM Bis-Tris and 0.2% dodecyl maltoside and incubated on ice for 30 min. The insoluble material was pelleted by centrifugation at 100,000 x g for 30 min and the supernatant was mixed with blue native loading buffer (750 mM aminocaproic acid, 50 mM Bis-Tris, 0.5 mM EDTA and 5% Serva Blue G) and separated on either a 5–16% (when probed for CcO or complex II) or a 5–13% native gradient gel (when probed for complex I, III or V). The gels were run at 100 V, initially with cathode buffer containing the blue dye. When the dye front reached the middle of the gel the buffer was replaced by clear cathode buffer. Electrophoresis was carried out until the blue dye reached the end of the gel. Protein was transferred to PVDF membrane (20 mA, 30 min) and the membranes were probed with specified antibodies.

### Electron transport chain complex assays

Assays for complex I, complex II–III and CcO were performed as described by Birch-Machin and Turnbull [31] using a Cary 1E UV-Vis Spectrophotometer. Briefly, complex I activity (NADH: ubiquinone oxidoreductase) was measured by incubating 25 μg of freeze-thawed mitochondrial extract in 1 ml of assay medium (25 mM potassium phosphate, pH 7.4, 5 mM MgCl_2_, 2 mM NaCN, 2.5 mg/ml BSA, 13 mM NADH, 65 μM ubiquinone and 2 μg/ml antimycin A) and measuring the decrease in absorbance at 340 nm due to NADH oxidation. Complex II–III activity (succinate–cytochrome *c* reductase) was measured by incubating 25 μg of freeze-thawed mitochondrial extract in 1 ml of assay medium (25 mM potassium phosphate, pH 7.4, 2 mM NaCN, 20 mM succinate, 2 μg/ml rotenone and 37.5 μM oxidized cytochrome *c*) and measuring the increase in absorbance at 550 nm due to cytochrome *c* reduction. CcO activity was measured by incubating 1 μg of freeze-thawed mitochondrial extract in 1 ml of assay medium (25 mM potassium phosphate, pH 7.4, 0.45 mM dodecyl maltoside and 15 mM reduced cytochrome *c*) and measuring the decrease in absorbance at 550 nm due to cytochrome *c* oxidation. Mitochondrial Aconitase activity was assayed as described before [32]. Briefly, 50 μg of mitochondrial protein was added to the assay buffer containing 50 mM Tris pH 7.4, 0.2 mM NADP, 0.6 mM MnCl_2_, and 30 mM sodium citrate. The reaction was followed at 340nm and the result represented as µmols of NADPH formed. 

### Measurements of oxygen consumption rate

The XF24 Extracellular Flux Analyzer (Seahorse Bioscience, Billerica, MA) was used to measure mitochondrial respiration to assess the effect of hypoxia on the electron transport chain complexes. Maximum mitochondrial respiratory capacity is an indicator of the integrity and functionality of the respiratory chain complexes. It was measured by uncoupling mitochondrial respiration by treating cells with 2,3-dinitrophenol (100µM). RAW 264.7 cells were maintained under normoxia or under hypoxia for 12h as explained above with or without the inhibitors. After hypoxia, the compounds were washed off with fresh medium and the cells were incubated for 3h in XF24 medium under normoxia. Finally, the cells were seeded in XF24 microplates (100,000 cells per well) and centrifuged at 1300 rpm for 8 min at room temperature. Both basal respiration and uncoupled respiration were measured in normoxic conditions. At the end of measurement, total protein was estimated from each well of the plate by BCA method (Pierce, USA) and used to normalize the oxygen consumption rate values. Maximum Oxygen consumption rates were calculated by subtracting oxygen consumption rate measured after addition of Rotenone and Antimycin (1µM each) from uncoupled respiration measured after addition of 2,3-dinitrophenol. 

### Low-temperature EPR (electron paramagnetic resonance) of control and ischemic mouse hearts

Control and ischemic hearts were frozen in liquid nitrogen immediately after the experiment and stored at −70 °C until EPR measurements were performed. 

#### EPR measurements

The X-band EPR of cardiac tissues were recorded at liquid helium temperatures on a Bruker E500 ELEXYS spectrometer with 100 kHz field modulation, equipped with an Oxford Instrument ESR-9 helium flow cryostat and a DM-0101 cavity. For EPR measurements, frozen heart tissues from control and ischemia-reperfusion experiments were minced to appropriate sizes under liquid nitrogen, and then transferred to a 4 mm quartz EPR tube (Wilmad-Lab Glass, Buena, NJ). The heart tissue weight in each EPR experiment was about 0.3 g. The volume of the tissue sample in the EPR tube was always greater than the working zone of the cavity in each EPR experiment. The only variable was the packing factor (i.e., filling of EPR tubes with tissues). However, this tissue packing factor was not perceived to be a major problem, as reloading of tissues in the same EPR tube (with a different packing) resulted in EPR spectra with a 5% variation in signal intensity. Spectrometer conditions were as follows: microwave frequency, 9.635 GHz; modulation frequency, 100 kHz; modulation amplitude, 10 G; receiver gain, 85 dB; time constant, 0.01 s; conversion time, 0.08 s; sweep time, 83.9 s. EPR spectra were obtained over the temperature range 4-50 K using an incident microwave power of 5 mW and modulation amplitude of 10 G. The spectrometer was calibrated with the radical 1,1-diphenyl-2-picrylhydrazyl (DPPH) exhibiting an EPR signal centered at g = 2.0036.

### Measurement of Reactive Oxygen Species

ROS generation by normoxic and hypoxic cells was measured by Dichlorofluorescein fluorescence. Briefly, RAW 264.7 cells were maintained under normoxia or hypoxia in presence of N-Acetyl Cysteine or Mito-CP. At the end of hypoxia, cells were counted and plated in 96 well plates in phosphate buffered saline. DCFDA (1µM) was added to each well and incubated for 15 min. DCF fluorescence was measured in a Chameleon plate reader (Hidex, Finland) at Excitation 525nm and Emission 575nm. ROS generation in isolated mitochondria was measured using Amplex Red. Briefly, mitochondria (10µg) isolated from normoxic and hypoxic RAW 264.7 cells expressing wild type or S40A mutant CcO Vb was plated in 96 well plate in phosphate buffered saline containing 10mM Succinate. Amplex red (10 µM), 4U/ml Horseradish peroxidase and 40U/ml Superoxide dismutase were added and the reaction was incubated at 37°C for 15min. Fluorescence was measured in a Chameleon plate reader (Excitation 530nm, Emission 590nm).

### cAMP measurements

cAMP was measured in both total homogenate and isolated mitochondria using Direct EIA cAMP kit according to manufacturer’s protocol (Enzo life sciences, NY). For each measurement 10µg protein was used. Values represent average of triplicate assays from two separate experiments.

## Statistical Analysis

Data from the cultured macrophages and isolated heart studies are presented as means ± S.D. Differences between paired variables were determined using two-tailed Student's *t* tests for paired data *p* values <0.05 were considered statistically significant.

## Results

### Experimental systems

RAW 264.7 macrophages present an excellent cell system for studying hypoxia mediated changes for several reasons. First, they respond to hypoxia readily and express markers of hypoxic stress at high level [33–35]. Second, activation of macrophages is an indicator of inflammatory response in most tissue and in the ischemic injury in the myocardial tissue [34,35]. Thus, hypoxic response in macrophage can be an important indicator of damage to myocardial tissue during ischemic injury. Results obtained with RAW 264.7 macrophages were further confirmed using a mouse heart ischemia/reperfusion system.

### Effect of hypoxia on mitochondrial PKA activity

**Figure 1:**
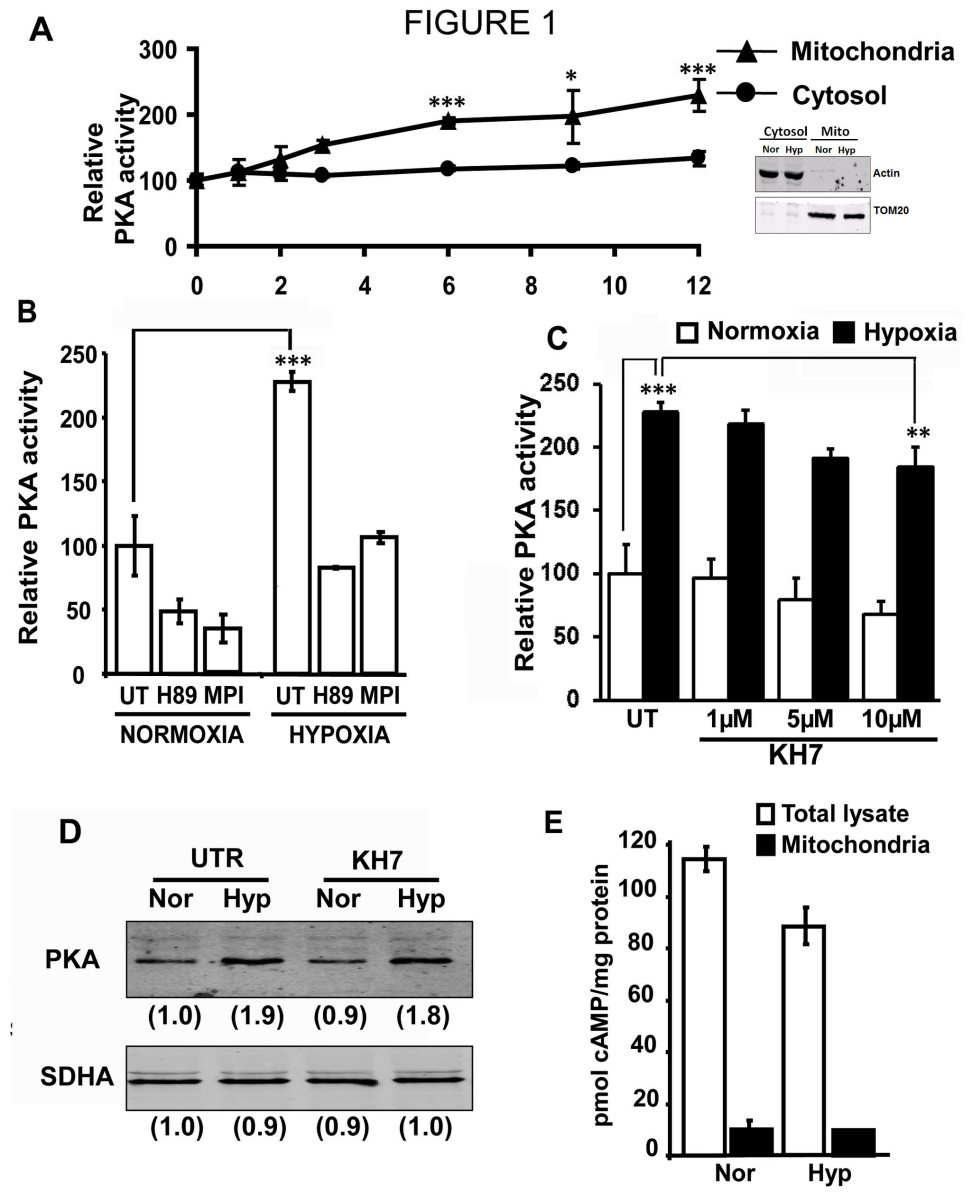
Hypoxia induces mitochondrial PKA activity in RAW 264.7 macrophages. A) PKA activity in isolated mitochondrial and cytosol of RAW 264.7 macrophages subjected to hypoxia for 0-12h. The activity of the corresponding normoxic fraction was taken as 100% activity. The inset shows immunoblot of representative mitochondrial and cytosolic fractions for cross contamination. Blot was probed with antibodies to Actin and TOM20 as markers of cytosol and mitochondria. 30µg of mitochondrial and cytosolic proteins were loaded in each well. B) PKA activity in mitochondria isolated from normoxic and hypoxic cells. H89 (1µM) and MPI (1µM) were added at the start of hypoxia (n=3) C) Effect of KH7 on hypoxia induced mitochondrial PKA activity. RAW 264.7 cells were treated with indicated concentrations of KH7 at the start of hypoxia (n=3) D) Immunoblots showing level of PKAα in mitochondria isolated from normoxic and hypoxic cells with or without KH7 (10µM) treatment. Values in parantheses underneath each blot are relative intensities of the bands. Blot is representative of two separate experiments. E) cAMP levels in 10µg of whole cell lysates and isolated mitochondria from normoxic and hypoxic cells. The difference in cAMP level between normoxic and hypoxic total lysates was not significant. *, p<0.01; **, p<0.005; ***,p<0.001.

Previous studies from our laboratory showed that PKA activity associated with mitochondria increased in cells subjected to hypoxia and hearts subjected to ischemia-reperfusion [10]. In this study we investigated the sub-mitochondrial location as well as the nature of PKA activated under hypoxia. For this purpose RAW 264.7 macrophages were subjected to 0-12h of hypoxia (1% O_2_) and PKA activity was measured in both isolated mitochondria and cytosol. As seen in Figure 1A, there was a time dependent increase in PKA activity in mitochondria up to 12h of hypoxia. The activity in the cytosolic fraction, on the other hand, did not significantly change during this period. The inset in Figure 1A shows that mitochondria used were relatively free of cytosolic contamination. This is important since the cytoplasmic PKA activity is about 10 fold higher than mitochondrial PKA activity. Figure 1B shows the effect of inhibitors on mitochondrial PKA activity. Two PKA inhibitors; H89, an isoquinolinesulfonamide and MPI, a specific peptide inhibitor were used. Mitochondrial PKA activity in both normoxic and hypoxic cells were inhibited by H89, and MPI (Figure 1B). However, KH7, a specific inhibitor of soluble adenylate cyclase had no significant effect on hypoxia mediated increase in mitochondrial PKA activity (Figure 1C). In addition, KH7 did not have any effect on the mitochondrial level of PKAα subunit in hypoxic cells (Figure 1D). Since cAMP is the known activator of PKA, we measured the level of cAMP in both cytosol and mitochondria of cells subjected to hypoxia. As shown in Figure 1E, cAMP level decreased by about 15% in total lysate from hypoxic cells, but was unchanged in mitochondria. These results suggest that increase in mitochondrial PKA activity under hypoxia is independent of cAMP.

### Increased mitochondrial localization of PKAα subunit under hypoxia

**Figure 2:**
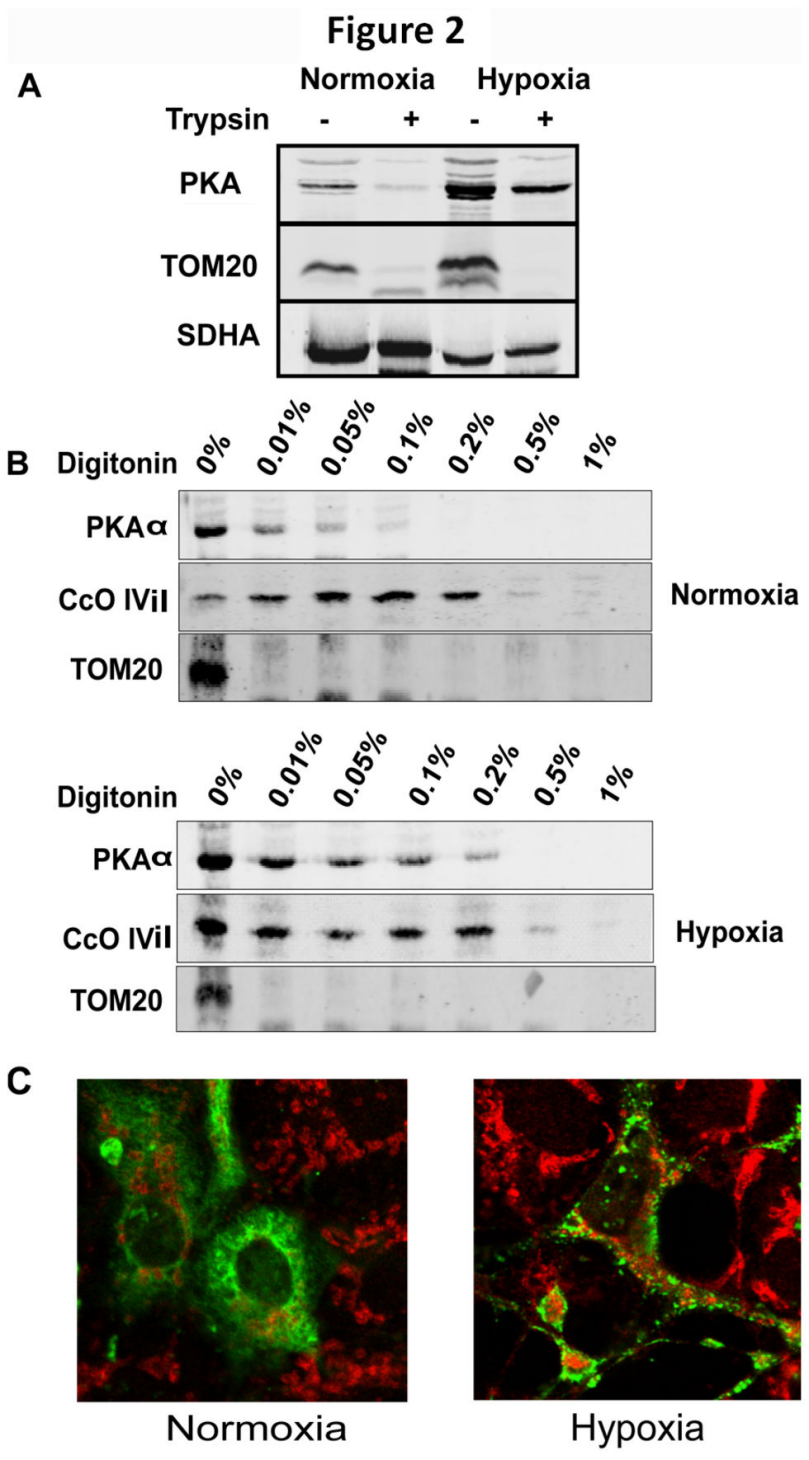
Hypoxia increases the intra mitochondrial pool of PKAα. A) Immunoblots of the PKAα subunit in mitochondria from normoxic and hypoxic macrophages. For trypsin treatment, mitochondria (100µg) were incubated with 15µg of trypsin at 4°C for 20 min. Tom20 and SDHA were used as outer and inner mitochondrial membrane markers, respectively. B) Mitochondria (100 µg) from normoxic and hypoxic cells were incubated in increasing amounts of digitonin (0-1% digitonin), in the presence of 15 µg Trypsin, as described in Materials and Methods. Mitochondrial pellets were separated by SDS PAGE and immunoblotted for PKAα, CcO IVi1 (inner membrane marker) and TOM20 (outer membrane marker). Upper panel: Immunoblots for normoxic cell mitochondria; lower panel: immunoblot for hypoxic cell mitochondria. The immunoblots are representative of three independent experiments. C) Immunofluorescence images of COS-7 cells transfected with cDNA for FLAG-tagged PKAα and grown under normoxic or hypoxic conditions. Cells were stained for FLAG (green) and CcO I (red) as described in Material and Methods.

It is known that PKAα and regulatory β subunits are anchored on AKAP proteins localized on the mitochondrial outer membrane, facing the cytosol [36–38] as well as mitochondrial inner membrane facing the matrix [39,40]. To understand the mechanism of activation of PKA under hypoxia we used multiple approaches to quantify the intra mitochondrial PKAα subunit under normoxic and hypoxic conditions. Figure 2A shows increased levels of PKAα in mitochondria of hypoxic cells. Further, to differentiate the PKA subunits associated with mitochondrial outer membrane, we incubated the mitochondria with trypsin as described in Materials and Methods. While most of the PKA associated with normoxic mitochondria was degraded by trypsin, substantial portion of PKAα was protected in mitochondria from cells subjected to hypoxia (Figure 2A). The full size images of the immunoblots are presented in Figure S1. 

To further ascertain the increased sequestration of PKAα in hypoxic mitochondria, intact mitochondria isolated from normoxic and hypoxic cells were permeabilized by incubating with increasing concentrations of digitonin. The reaction also included a small amount of trypsin to digest proteins that are released from mitochondria progressively with increasing digitonin concentrations, starting with the outer membrane to the inner membrane. At the end of the reaction samples were denatured and used for immunoblot analysis with antibodies for PKAα subunit. The CcO subunit IVi1 was used as a marker of inner mitochondrial membrane and TOM20 as marker for the outer mitochondrial membrane. Figure 2B, shows proteins sensitive and resistant to trypsin digestion following digitonin treatment. Mitochondria from normoxic cells (Top panel, first lane) not treated with digitonin and trypsin show full complement of PKAα, CcO IVi1 and TOM20. At all concentrations of digitonin (0.01 to 1%) and trypsin, TOM20 was degraded, while PKAα was protected up to 0.05% of digitonin. CcO IVi1 which is an inner membrane integral protein was resistant to trypsin digestion up to 0.2% digitonin at which point the inner membrane becomes permeable. In hypoxic cell mitochondria, however, PKAα subunit showed resistance to trypsin digestion up to 0.2% digitonin concentration, similar to CcO IVi1 subunit (Figure 2B, bottom panel). The full size images of the immunoblots are presented in Figure S2. At this concentration digitonin essentially disrupts mitochondrial outer membrane [41] making any intermembrane space PKA vulnerable to trypsin digestion. Thus the results suggest that higher level of PKAα subunit is sequestered in the mitochondrial inner membrane compartment under hypoxic conditions.

To confirm the altered distribution of PKAα subunit during hypoxia, we ectopically expressed tagged PKAα subunit in COS-7 cells and followed its subcellular distribution under normoxia and hypoxia through immunofluorescence microscopy. The confocal microscopy patterns in Figure 2C show that in normoxic cells (left panel) ectopically expressed PKAα is distributed throughout the cells with few cells showing immuno staining in mitochondria. In cells subjected to hypoxia, however, there was an increase in PKAα colocalization with mitochondria specific CcO I antibody (Figure 2C, right panel).These results together suggest that in normoxic cells PKAα is mostly distributed in the cytosol and on the mitochondrial outer membrane in a manner readily accessible to trypsin digestion. In hypoxic mitochondria, a significantly higher level of PKAα subunit was localized inside the mitochondrial inner membrane, and was not readily accessible to trypsin suggesting its localization on the matrix side. 

### Effect of antioxidants on mitochondrial PKA activation during hypoxia

**Figure 3:**
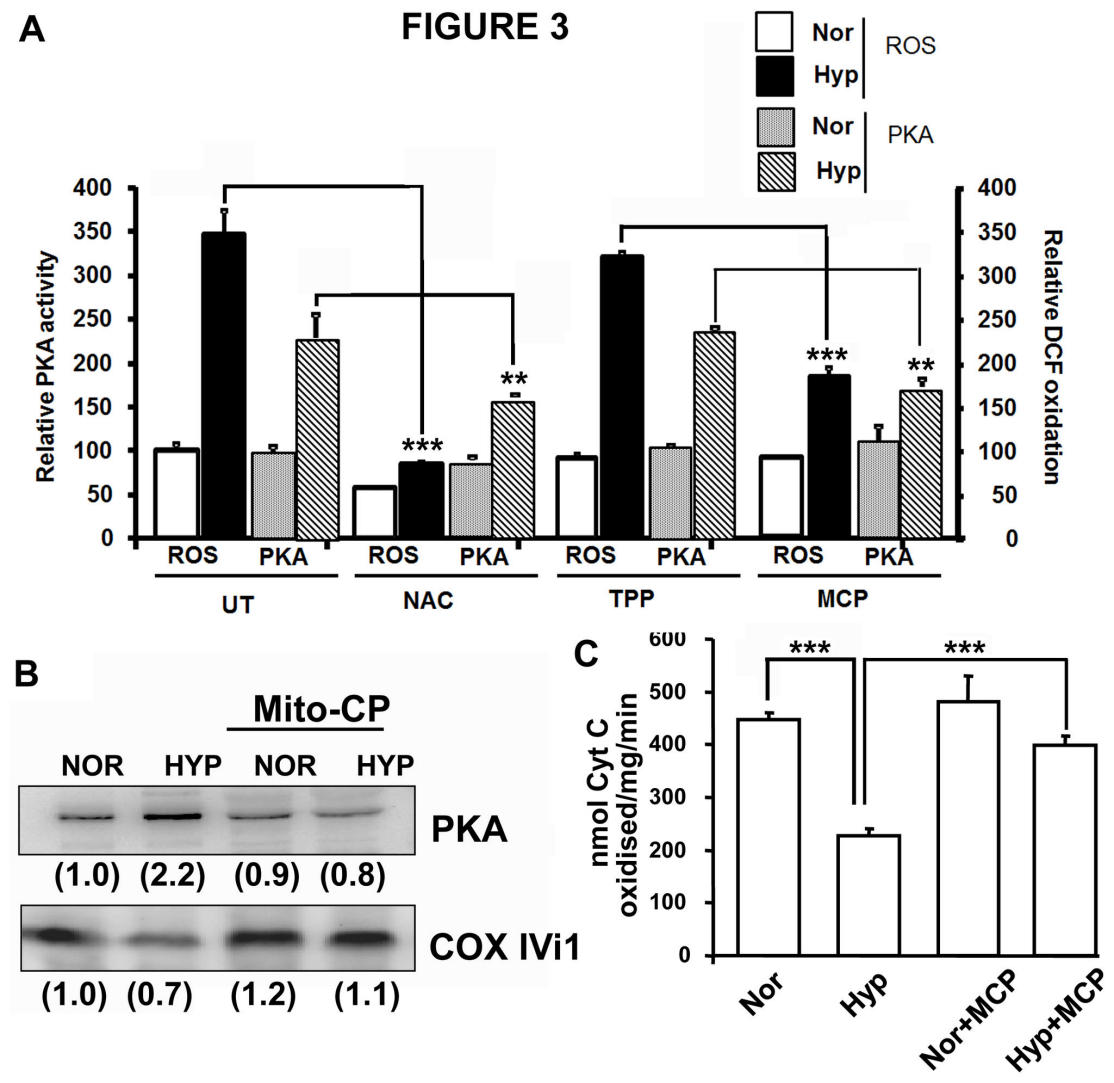
Effects of antioxidants on mitochondrial PKAα subunit level and activity. RAW 264.7 macrophages were subjected to hypoxia for 12h with or without addition of 1µM Mito-CP or 10mM N-Acetyl Cysteine. At the end of hypoxia, part of the cells was used for measuring ROS production by DCFDA oxidation and the remaining for mitochondria isolation. Protein was estimated by Lowry’s method. A) PKA activity and ROS production (n=3). After hypoxia cells were plated in 96 well plate in phosphate buffered saline and incubated with DCFDA (1µM) for 15 minutes. Fluorescence was measured at Excitation 525nm and Emission 575nm. Corresponding PKA activity was measured in 10µg of mitochondrial protein, B) PKAα protein level. 30µg of mitochondrial protein was separated on SDS PAGE and transferred to nitrocellulose membrane. PKAα and CcO IVi1 antibodies were used for immunoblotting. Relative band intensities are given in parantheses. The blot is representative of two separate experiments. C) Effect of Mito-CP on CcO activity under hypoxia. CcO activity was measured with 10µg of mitochondria (n=4). **, p<0.005.

Since hypoxia did not result in any increase in cAMP level either in cytosol or mitochondria and activation of PKA was unaffected by adenylate cyclase inhibitor, KH7, we investigated alternative mechanisms of activation of PKA. Oxidative stress and ROS has been implicated in the cAMP independent activation of PKA [42–44]. We therefore tested the effects of two different antioxidants on hypoxia modulated mitochondrial PKA activation. Figure 3A shows that both general antioxidant N-Acetyl Cysteine and mitochondria targeted Mito-CP not only reduced ROS levels, but also attenuated mitochondrial PKA activation in cells subjected to hypoxia. Treatment with the targeting vehicle TPP+ alone did not affect hypoxia mediated increases in ROS levels as well as mitochondrial PKA activity. Immunoblot in Figure 3B shows that the antioxidants also attenuated hypoxia mediated increase in the level of mitochondrial PKAα subunit. Decreased mitochondrial PKA activation was accompanied by significant protection of CcO activity under hypoxia (Figure 3C).These results suggest that activation of PKA under hypoxia is associated with increased ROS production. Inhibition of ROS and prevention of loss of CcO activity by mitochondria targeted antioxidant suggests that the activation is predominantly, if not exclusively because of mitochondrially generated ROS. 

### Effect of hypoxia induced PKA activity on mitochondrial respiration

**Figure 4:**
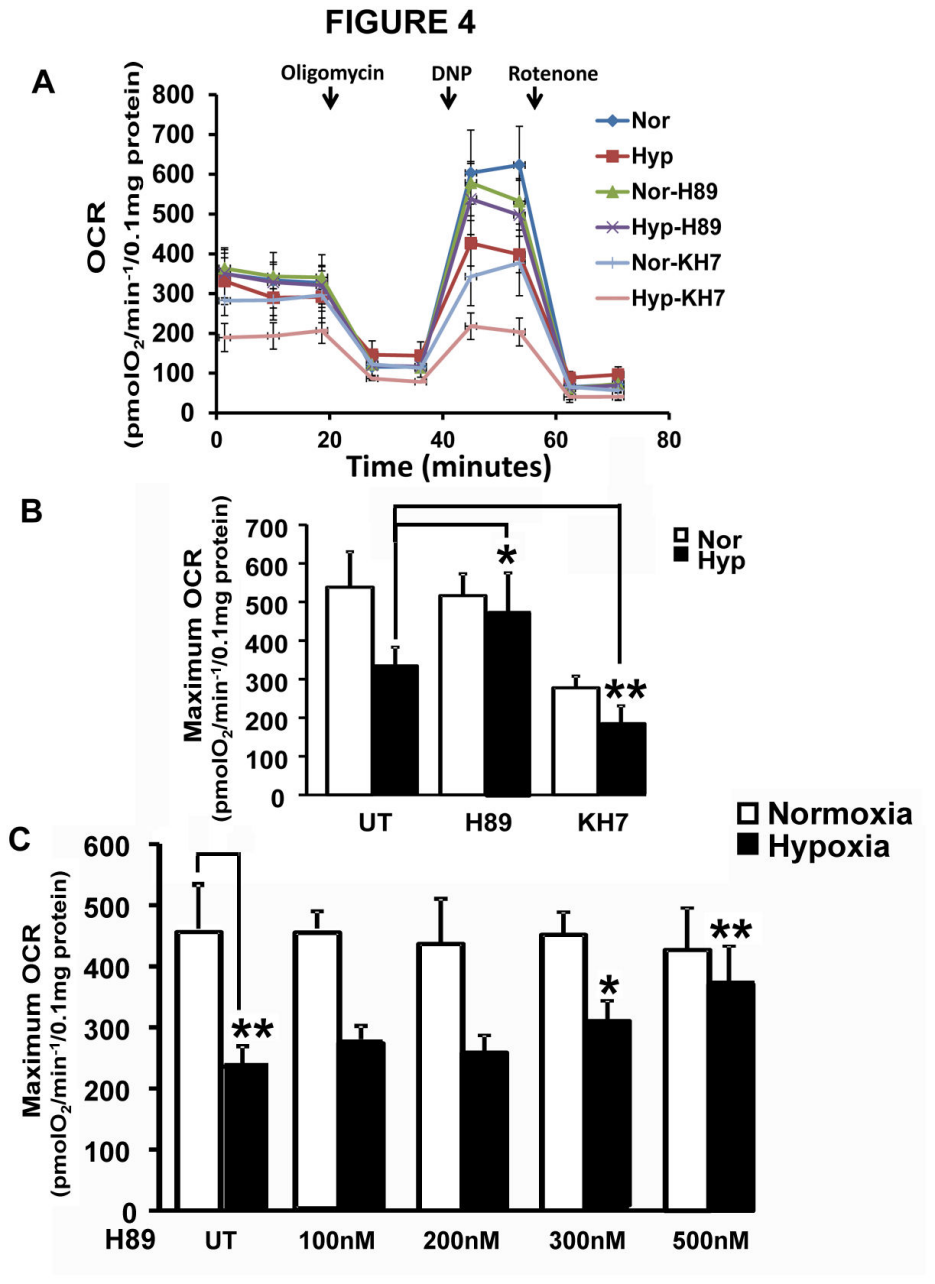
Role of hypoxia induced PKA on mitochondrial respiratory capacity. Macrophages grown under normoxic or hypoxic conditions in the presence or absence of H89 or KH7 were used for measuring Oxygen consumption in a Seahorse XF extracellular flux analyzer. Cells were maintained under either hypoxia or normoxia in presence of inhibitors for 12h. Before measurement of respiration, the cells were washed with fresh medium without inhibitors and plated on XF24 plates and incubated for additional 3h. A) Respiration measurement in presence of H89 (1µM) and KH7 (10µM) is shown. DNP=2,3 dinitrophenol. Rot/AA=Rotenone and Antimycin. This is a representative profile of three replicates B) The maximum OCR of cells treated with H89 or KH7 under hypoxia. Maximum OCR was calculated by subtracting residual OCR after adding Rotenone and Antimycin from OCR measured after uncoupling with 100µM DNP. The values were normalized to 100µg of total cellular protein. C) Maximum OCR in cells treated with different concentration of H89. Maximum OCR calculation and normalization were as described for B). Protection against loss of respiratory capacity by H89 was significant at 300nM and 500nM as indicated, compared to untreated hypoxic control. The values were derived from three separate experiments.*, p<0.05;**, p<0.005.

RAW 264.7 cells were treated with PKA modulators for the duration of growth under hypoxia or normoxia. The effect of PKA inhibition during hypoxia on mitochondrial respiratory capacity was assessed using Seahorse respirometer. Figure 4A shows the oxygen consumption rates of cells treated with or without H89 and adenylate cyclase inhibitor KH7 in the presence of specific respiratory chain inhibitors. Since PKA mediated phosphorylation has been shown to be an important mediator of electron transport chain complexes and respiration under normal physiological condition [22,45], H89 and KH7 were washed off 3h before respiration measurements following hypoxia. The respiratory pattern in Figure 4A and maximum OCR in Figure 4B show no significant effect of H89 under normoxia. However, H89 reversed the hypoxia mediated decline in OCR. KH7, on the other hand, showed marked inhibition of respiration in both normoxic and hypoxic cells. We observed substantial cell death in cultures treated with KH7, and we believe that the respiratory inhibition is probably due to unknown toxic effects of KH7 on RAW 264.7 cells. Figure 4C shows the concentration dependent effect of H89 on maximum OCR in hypoxic and normoxic cells. It is seen that at concentrations from 100nM to 500nM of H89, we observed less than 10% decrease in respiration under normoxia. Under hypoxic conditions, however, there is a concentration dependent increase in oxygen consumption rate suggesting significant protection against loss of respiratory capacity under hypoxia. These results clearly suggest that inhibition of PKA activity during hypoxia has a significant protective effect on mitochondrial respiratory capacity. 

### Phosphorylation resistant mutation prevents loss of CcO subunit Vb under hypoxia

**Figure 5:**
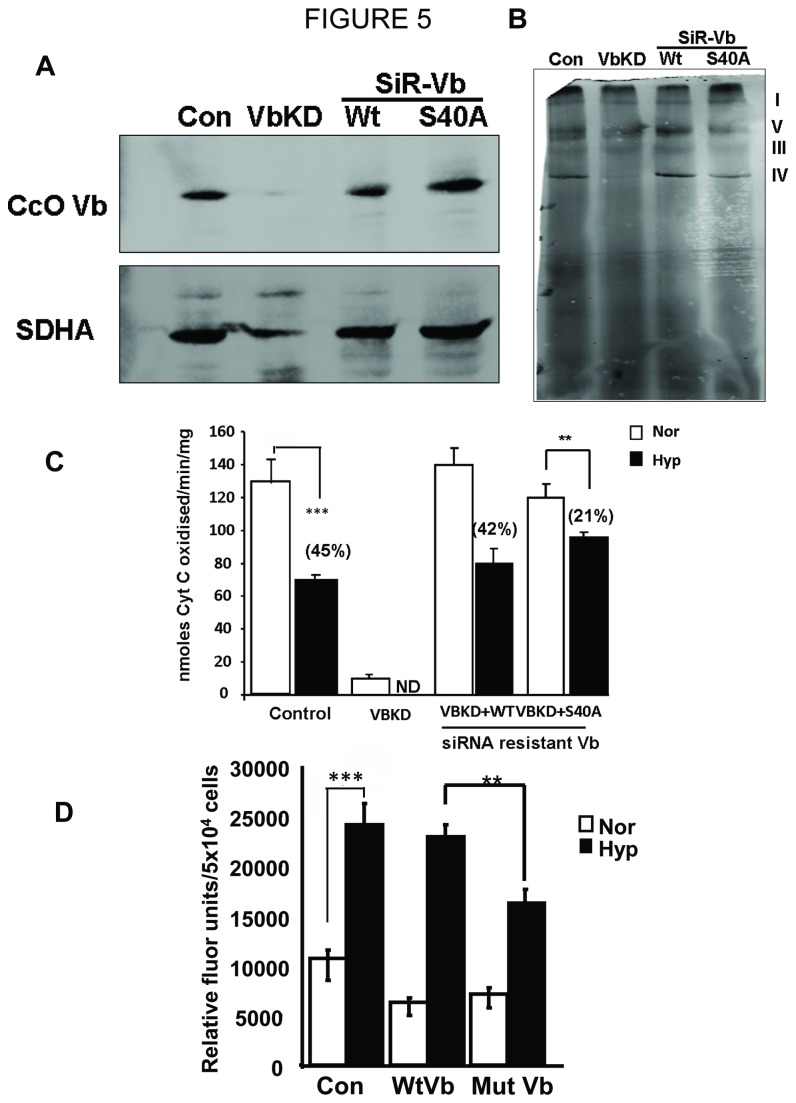
Reconstitution with phosphorylation resistant CcO Vb subunit attenuates hypoxia induced CcO dysfunction and ROS production. siRNA resistant (SiR) - wild type (WT) or S40A mutant CcO Vb were expressed in RAW 264.7 macrophages with CcO Vb knockdown (VbKD). A) Immunoblot of cell lysates showing CcO Vb levels in control, CcO Vb knockdown (VbKD) and si-RNA resistant CcO Vb expressing VbKD cells. 30µg of mitochondrial protein was separated on SDS PAGE and transferred to nitrocellulose membrane. Blots were stained with CcO Vb and SDHA antibodies. B) Blue Native PAGE performed with mitochondrial proteins from all cell types. 150µg of mitochondria from each sample was solubilized with Lauryl maltoside as described in Materials and Methods. Electrophoresis was carried out in a 6-13% gradient gel. Gel was destained to remove excess Coomassie stain and the bands were imaged in a scanner. C&D) Control, VbKD and SiR-wild type and S40A mutant cells were maintained under either normoxia or hypoxia. Mitochondria were isolated and proteins (25µg each) were used to measure CcO activity (C) and ROS production by Amplex red oxidation (D). n=4. **, p<0.005; ***, p<0.001.

Our previous studies showed that under both hypoxia and alcohol toxicity CcO subunit Vb was one of the three subunits reduced as a function of time [25,46]. To assess the role of phosphorylation on the selective degradation of Vb, we first generated a macrophage cell line stably expressing siRNA against CcO subunit Vb (VbKD). We then expressed either wild type (WT CcO Vb) or phosphorylation resistant mutant (S40A-CcO Vb) of the subunit in the VbKD cells. The CcO Vb cDNA contained degenerate codons in the siRNA target region to prevent siRNA mediated degradation in VbKD cells. Immunoblot in Figure 5A shows that the level of subunit Vb was markedly reduced in siRNA expressing cells (VbKD), which was restored in cells expressing the siRNA resistant wild type or S40A mutant cDNA. Figure 5B shows the Blue Native gel profiles of control, Vb knocked down cells and cells reconstituted with WT or S40A mutant CcO Vb subunits. It is seen that CcO complex was markedly reduced in Vb knocked down cells which was restored in cells reconstituted with WT or S40A mutant CcO Vb subunits. The effect of hypoxia on CcO activities of mitochondrial preparations from these cells is presented in Figure 5C. The CcO activity was reduced by about 45% in control cells exposed to hypoxia. In Vb knocked down cells, the basal activity was reduced by more than 95%. Reconstitution with either wild type or S40A mutant CcO Vb nearly completely restores CcO activity in these cells. Notably, in cells reconstituted with WT subunit Vb, hypoxia reduced CcO activity by 42%, while cells reconstituted with phosphorylation resistant S40A subunit showed only 21% loss of CcO activity under hypoxia (Figure 5C). It is also seen that under hypoxic conditions, the ROS production in cells reconstituted with WT CcO Vb was similar to the control cells. In cells reconstituted with S40A mutant subunit, however, there was a significant decrease in ROS production which was constituent with the extent of resistance to hypoxia induced subunit degradation (Figure 5D). These results suggest that the S40A mutant protein is relatively more resistant to hypoxia mediated damage and support our hypothesis that phosphorylation of subunits is responsible for hypoxia mediated loss of CcO activity. 

### Effect of mitochondrial PKA modulation on myocardial Ischemia/Reperfusion injury

**Figure 6:**
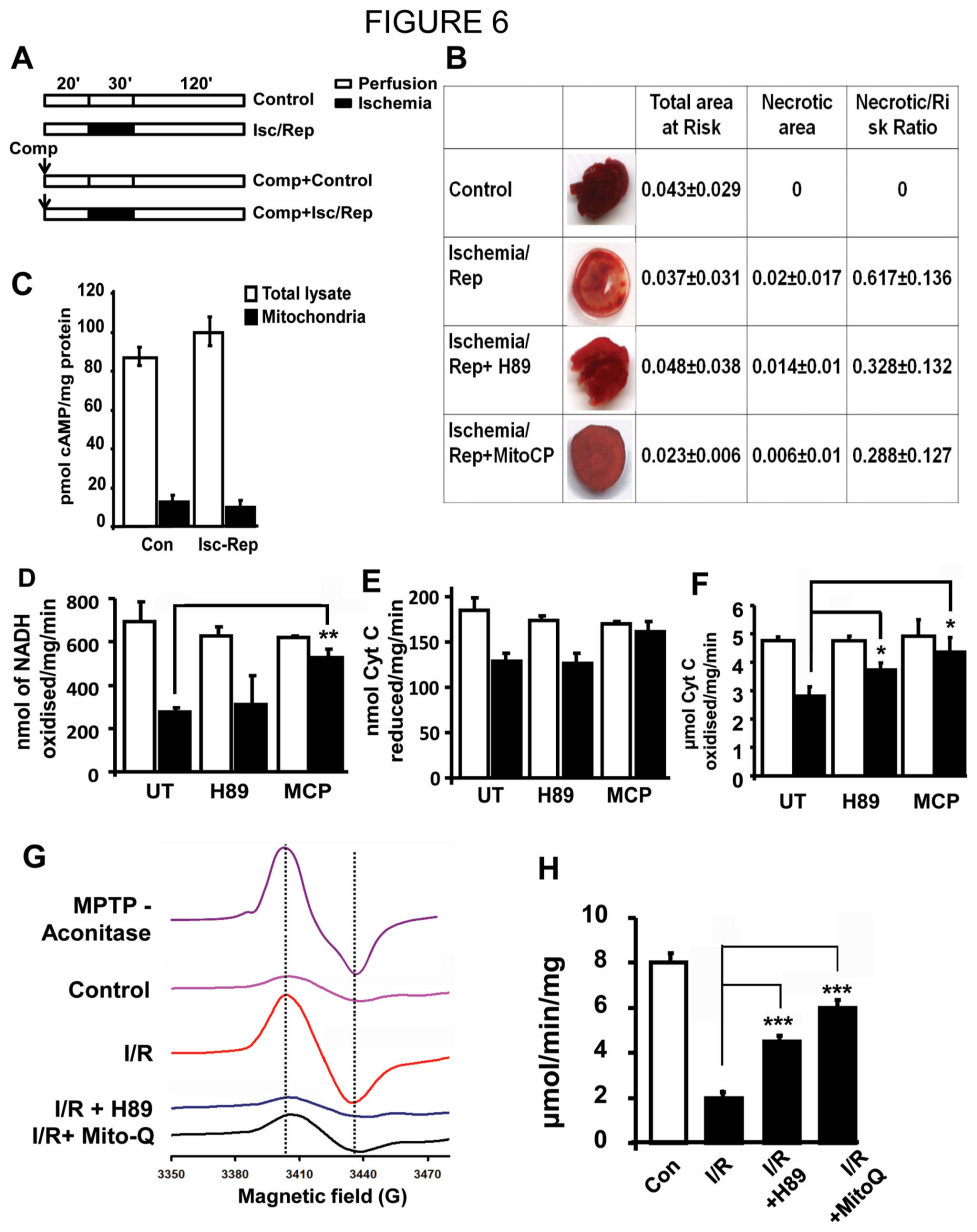
PKA inhibitor and mitochondrial antioxidant attenuate ischemia-reperfusion injury in perfused mouse heart. A) Schematic drawing showing the protocol of ischemia-reperfusion used for each group. B) Myocardial ventricular volumes (cm^3^) of mouse hearts after 30 min ischemia and 120 min reperfusion. Insets show representative photographs of midventricular myocardium after staining with 1% triphenyltetrazolium chloride solution to delineate the necrotic zone. For treatments, H89 (1µM) (n=6) or Mito-CP (1µM) (n=3) were maintained in the perfusion medium throughout the duration of the experiment. C) cAMP levels in 10µg of total homogenate and isolated mitochondria from control and ischemia-reperfused mouse heart tissues. D-F) Mitochondria were isolated from non-ischemic, ischemia-reperfused and H89 or Mito-Q preconditioned heart and used for measuring Complex I (D), III (E), IV (F) and Aconitase (H) activities (n=3). Protein used was as follows: Complex I and III, 25µg, Complex IV, 1µg and Aconitase, 30µg G) Low temperature EPR spectra of non-ischemic hearts and hearts subjected to ischemia reperfusion with or without preconditioning with H89 or Mito-*Q*. *Spectrum* from MPTP treated brain slice is shown as positive control for [3Fe-4S]^+^ aconitase. Conditions of spectroscopy: microwave power and frequency, 5 mW and 9.635 GHz; modulation amplitude and frequency, 10 G and 100 kHz; time constant, 0.01 s; temperature range, 4-50 K. *, p<0.01; **, p<0.005.

In order to extend our findings on the role of PKA in hypoxia in cell culture model to hypoxic conditions found in pathologies, we tested the effect of PKA modulators on myocardial ischemia-reperfusion injury. The infarct size resulting from temporary global ischemia and 2 hours of reperfusion was measured in mouse hearts perfused with PKA modulators. In untreated ischemia-reperfused hearts, the infarct size amounted to 61.7 % of the total area at risk (Figure 6B). The hearts that were perfused with H89 showed nearly 50% decrease in infarct size, suggesting protective effect of PKA inhibition during ischemia. Pre-perfusion with Mito-CP, a mitochondria targeted antioxidant resulted in nearly 60% reduction in necrotic tissue compared to untreated ischemic heart. The level of protection was comparable with that of H89 treatment. Consistent with our results with hypoxic cell mitochondria, the mitochondrial cAMP levels in ischemic hearts was not increased. The total tissue content of cAMP was increased marginally (Figure 6C). 

Activities of respiratory complexes and aconitase were measured in the heart tissues. Figure 6D shows that the rotenone sensitive complex I activity was inhibited by about 60% in ischemia-reperfused mouse hearts. Treatment with H89 only moderately restored the activity. The ubiquinol:ferrocytochrome c oxidoreductase activity (complex III) was inhibited by only about 20% during ischemia that was not protected significantly by H89 (Figure 6E). As seen in Figure 6F, ischemia-reperfusion decreased CcO activity in both cases by about 50%. PKA inhibitor, H89 prevented the loss of CcO activity to near control levels. 

Aconitase is an important marker of oxidative stress that has been shown to be oxidatively damaged under mitochondrial stress [47]. Oxidative damage to aconitase was prevented by PKA inhibition during ischemia-reperfusion as shown in Figures 6G and 6H. We ascertained the protection against damage to aconitase metal centers by low temperature EPR study. Figure 6G shows the profile of low temperature EPR from ischemic heart tissues subjected to different treatments. MPTP was used as a positive control to oxidatively damage aconitase. As shown, hearts subjected to Ischemia-reperfusion showed the characteristic peak that corresponds to oxidized metal center [3Fe-4S]^+^ of aconitase. Control hearts, on the other hand, did not show significant damage to metal centers. Importantly, preconditioning with H89 or Mito-Q significantly reduces the formation of oxidized aconitase in ischemia-reperfused hearts. Figure 6E shows aconitase activity in mouse hearts subjected to the same conditions as for low temperature EPR. As seen, aconitase activity was reduced by 75% of control levels in ischemia-reperfused hearts. Preconditioning with either H89 or Mito-CP, reverted the loss of aconitase activity significantly.

## Discussion

Protein kinases have long been shown to play roles in various aspects of mitochondrial biogenesis and function [11,48,49]. PKA mediated phosphorylation has been shown to activate mitochondrial TOM receptors in Yeast, thus directly regulating mitochondrial biogenesis [50]. Phosphorylation of electron transfer complexes and other mitochondrial proteins are examples of protein kinase dependent regulation of mitochondrial function [13,16,51–53]. In addition to regulating physiological functions, protein kinases have been implicated in a wide variety of intracellular signaling during ischemia-reperfusion injury and cellular hypoxia, responsible for both protective and harmful effects [18,54,55]. PKC delta has been shown to be activated by I/R and translocates to mitochondria where it both decreases ATP generation and causes cytochrome C release [55]. On the other hand, PKC epsilon, has a cardio protective function against I/R injury, and is known to prevent membrane permeability transition [18]. Similarly several lines of study have shown that GSK-3β activity determines myocardial vulnerability to reperfusion-injury [54]. 

PKA signaling in hypoxia has been studied by our lab and others in different model systems. In bone cells and bovine aortic endothelial cells, apparent PKA activity was shown to rapidly increase with decreasing oxygen concentration [56]. In PC-12 cells, hypoxic induction of PKA was shown to repress HIF-1α activity [57]. In A549 lung carcinoma cells, long term hypoxia induced PKA activity and was shown to be responsible for Epithelial Mesenchymal Transition [58]. PKA activity within mitochondria has been reported in different species [10,40,59–61]. CcO, the terminal oxidase of the electron transport chain has been shown to be one of the important targets of PKA [9,10,22,23]. Initially, Kadenbach’s laboratory showed that PKA mediated phosphorylation in vitro inhibited CcO activity by turning on allosteric inhibition by ATP [23]. More recent studies by Manfredi’s group showed that a mitochondrial soluble adenylate cyclase and cAMP dependent protein kinase under normoxic condition activated CcO by phosphorylation, suggesting positive modulatory effects under normal growth conditions [22,45]. In this paper we show that hypoxia and myocardial ischemia induce a novel mitochondrial PKA activity that appears to be independent of mitochondrial cAMP but dependent on ROS production. 

Our previous studies have shown that CcO subunits I, IVi1 and Vb are phosphorylated under hypoxia or myocardial ischemia-reperfusion [10]. Further immunoprecipitation of whole complex and immunoblot analysis on high resolution gels showed that these three subunits were selectively degraded, while the levels of other subunits remained unaltered [10]. The phosphorylation and subunit degradation was prevented by H89 and MPI, a peptide inhibitor of PKA. Although H89 can inhibit other kinases at high concentration, MPI is highly specific for PKA. Since the phosphorylation sites we identified [9] were only partially consensus for PKA mediated phosphorylation, it is likely that a PKA regulated kinase, rather than PKA itself, phosphorylates these sites. An alternate possibility is that these sites have a higher Kd for PKA association and a higher steady state level of mitochondrial PKA, such as that occurs under hypoxic condition, is necessary for phosphorylation.

Increased mitochondrial PKAα levels and activity were reported in rabbit hearts subjected to ischemia-reperfusion injury [10]. In this paper we show that (Figure 6C), this increased PKA activity does not accompany any increase in mitochondrial cAMP level. Digitonin fractionation of isolated mitochondria and treatment with trypsin show higher levels of PKAα subunit in mitochondrial matrix compartment in response to hypoxia (Figures 1 and 2). Notably, we observed an increase only in the mitochondrial PKA activity under hypoxia with no significant increase in the cytosolic PKA activity up to 12h of hypoxia. Our results also show different subcellular distribution of ectopically expressed/tagged PKAα subunit under hypoxia. These results suggest that increased mitochondrial PKAα under hypoxia is probably due to translocation of cytosolic PKAα to mitochondria. Presently, the precise mechanism of translocation of cytosolic PKAα to mitochondria remains unclear. However, it is known that a number of ER targeted and cytosolic proteins are bimodally targeted to mitochondria by virtue of the chimeric signals they carry [62–64]. 

The increase in mitochondrial PKAα subunit level under hypoxia is prevented by the antioxidants N-acetyl cysteine and Mito-CP, which suggests a direct role of ROS in the activation (Figure 3). Previously we have shown that, PKA inhibitors, H89 and PKI attenuated the loss of CcO activity in cells subjected to hypoxia [9,10]. In the current study we observed that Mito-CP also attenuated the loss of CcO activity in cells subjected to hypoxia, possibly by preventing the activation of PKA. Similarly, both mitochondria targeted antioxidant MitoQ and H89 markedly reduced the infarct size in myocardial ischemia/reperfusion injury. Thus, the PKA mediated functional changes in CcO and mitochondrial dysfunction is an important factor in inducing ischemia/reperfusion damage to the myocardial tissue.

Previously we identified the ischemia/reperfusion induced phosphorylation site to Ser40 of CcO Vb subunit [9]. In this study we sought direct proof for the involvement of this site in hypoxia mediated loss of CcO activity using cells reconstituted with phosphorylation resistant CcO Vb subunit. Our results show that cells expressing S40A CcO Vb is relatively resistant to hypoxia induced loss of the subunit and also activity. These cells also showed lower propensity for ROS production suggesting a link between loss of CcO activity and ROS production under hypoxia. These results along with our previous studies provide direct evidence for the role of subunit phosphorylation in loss of CcO activity and that S40 of CcO Vb is an important target for hypoxia induced phosphorylation [9,10]. The phosphorylation sites of subunits IVi1 and Vb that we identified are part of a loop out structure exposed to the matrix side [9,65] and were shown to be phosphorylated under ischemia/reperfusion by LC-MS-MS analysis of phosphopeptides [9]. The mutational analysis presented in this study provides further proof for the functional significance of phosphorylation at these sites.

Highly coupled mitochondria with steep trans membrane potential have been shown to generate high levels of ROS mostly through catalytic activities of complex I and reverse electron transfer [66,67]. Studies from our group and others have shown that dysfunctional mitochondria with disrupted trans membrane potential also produce ROS under pathological conditions like ischemia/reperfusion [10] and hypoxia [68,69] and Figure 5 of present study. We postulate that increased ROS production associated with loss of CcO activity is either through accumulated reduced intermediates of the electron transport chain or more likely due to disrupted supramolecular respirosome complexes [70]. 

In summary, we describe a novel mechanism of mitochondrial PKA activation under hypoxia and ischemia/reperfusion. This PKA is distinctly different from the relatively low levels of basal PKA activity reported under normoxic conditions for the following reasons: 1. the stress induced PKA is modulated by ROS, 2. its target sites are distinctly different from sites reported under normoxic conditions, 3. The PKA activity induced under hypoxic condition is not inhibited by KH7, an inhibitor of soluble adenylate kinase, 4. Phosphorylation of CcO by stress induced PKA induces subunit degradation and CcO dysfunction rather than activation of CcO reported under normoxic conditions.

## Supporting Information

Figure S1 **Full images of immunoblots presented in Figure 2A.** The experimental details are given in materials and methods and Figure 2A.
(PPTX)

Figure S2 **Full images of immunoblots presented in Figure 2B.** NS=Non specific. The experimental details are given in materials and methods and Figure 2B.
(PPTX)
