# Supplementary figures and images for: Oxidative Stress Induced Mitochondrial Protein Kinase A Mediates Cytochrome C Oxidase Dysfunction
Source: PLoS One. 2013 Oct 10;8(10):e77129. doi: 10.1371/journal.pone.0077129 (PMC3795003; doi:10.1371/journal.pone.0077129)

## Slide 1
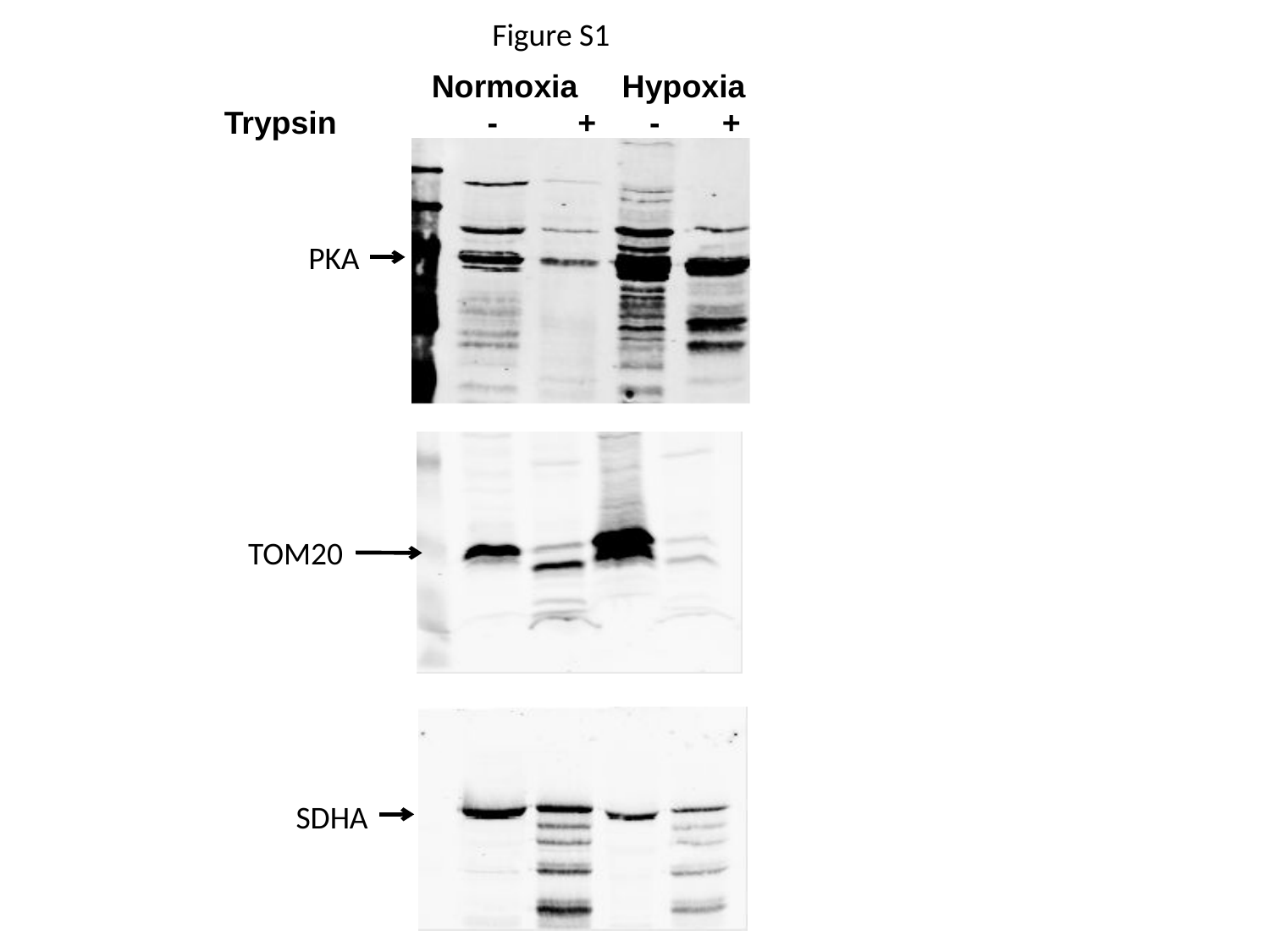

Figure S1
Normoxia Hypoxia
Trypsin - + - +
PKA
TOM20
SDHA

Supplement: Figure S1 — Full images of immunoblots presented in Figure 2A. The experimental details are given in materials and methods and Figure 2A. (PPTX) [file pone.0077129.s001.pptx]

## Slide 1
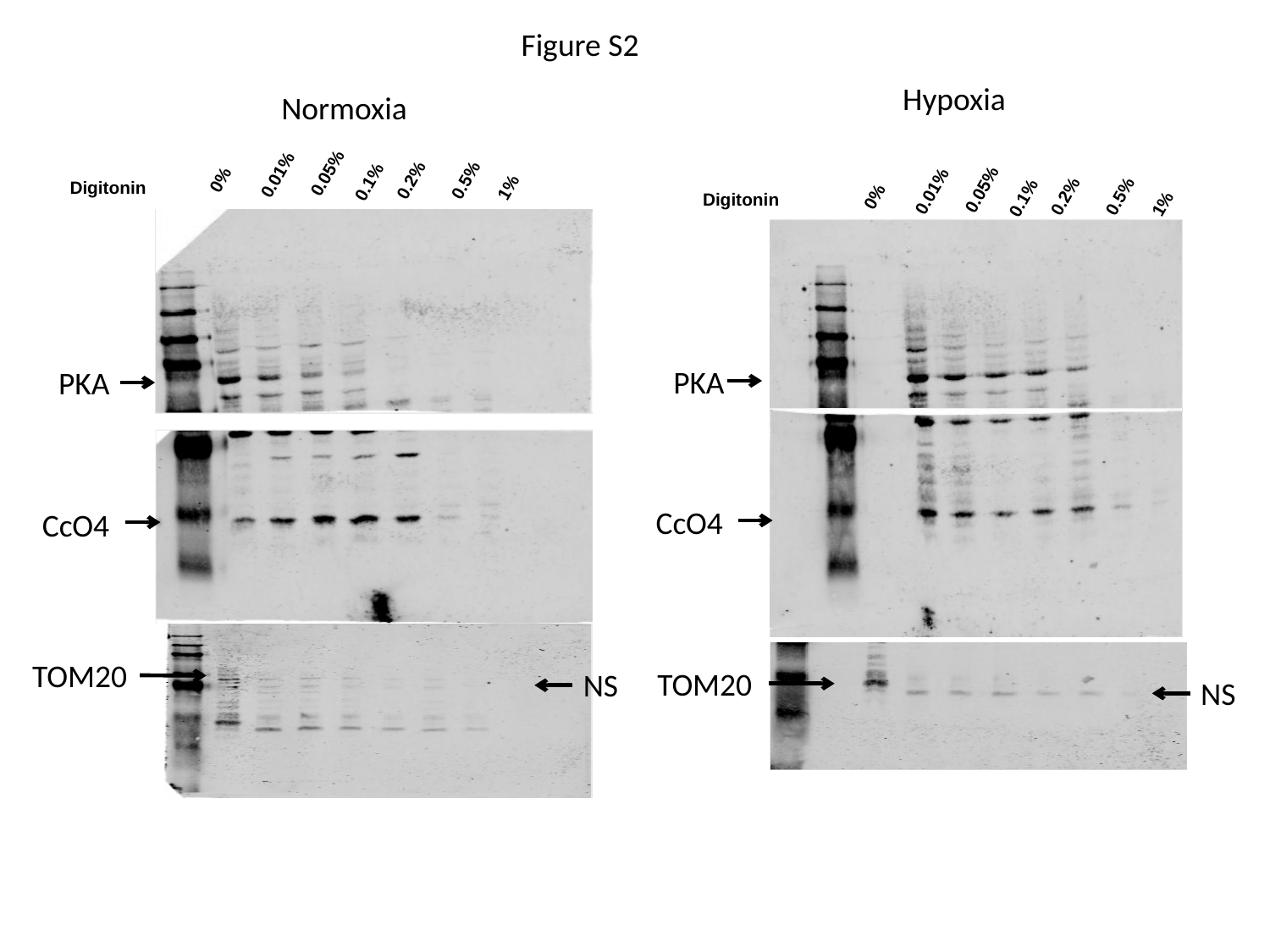

Figure S2
Hypoxia
Normoxia
0.05%
0.01%
0.5%
0%
0.2%
0.1%
Digitonin
1%
0.05%
0.01%
0.5%
0%
0.2%
0.1%
Digitonin
1%
PKA
PKA
CcO4
CcO4
TOM20
TOM20
NS
NS

Supplement: Figure S2 — Full images of immunoblots presented in Figure 2B. NS=Non specific. The experimental details are given in materials and methods and Figure 2B. (PPTX) [file pone.0077129.s002.pptx]
